# Modeling riboflavin transporter deficiency type 2: from iPSC-derived motoneurons to iPSC-derived astrocytes

**DOI:** 10.3389/fncel.2024.1440555

**Published:** 2024-07-24

**Authors:** Valentina Magliocca, Angela Lanciotti, Elena Ambrosini, Lorena Travaglini, Veronica D’Ezio, Valentina D’Oria, Stefania Petrini, Michela Catteruccia, Keith Massey, Marco Tartaglia, Enrico Bertini, Tiziana Persichini, Claudia Compagnucci

**Affiliations:** ^1^Molecular Genetics and Functional Genomics, Ospedale Pediatrico Bambino Gesù, IRCCS, Rome, Italy; ^2^Department of Science, University “Roma Tre”, Rome, Italy; ^3^Department of Neuroscience, Istituto Superiore di Sanità, Rome, Italy; ^4^Unit of Translational Cytogenetic Research, Laboratory of Medical Genetics, Bambino Gesù Children's Hospital, IRCCS, Rome, Italy; ^5^Confocal Microscopy Core Facility, Research Laboratories, Bambino Gesù Children's Hospital, IRCCS, Rome, Italy; ^6^Unit of Neuromuscular and Neurodegenerative Disorders, Translational Pediatrics and Clinical Genetics, Ospedale Pediatrico Bambino Gesù, IRCCS, Rome, Italy; ^7^Cure RTD Foundation, Calgary, AB, Canada

**Keywords:** neurodegenerative autosomal recessive disease, riboflavin transporter deficiency, redox state, induced pluripotent stem cells, astrocytes, motoneurons, *in vitro* disease modeling

## Abstract

**Introduction:**

Riboflavin transporter deficiency type 2 (RTD2) is a rare neurodegenerative autosomal recessive disease caused by mutations in the SLC52A2 gene encoding the riboflavin transporters, RFVT2. Riboflavin (Rf) is the precursor of FAD (flavin adenine dinucleotide) and FMN (flavin mononucleotide), which are involved in different redox reactions, including the energetic metabolism processes occurring in mitochondria. To date, human induced pluripotent stem cells (iPSCs) have given the opportunity to characterize RTD2 motoneurons, which reflect the most affected cell type. Previous works have demonstrated mitochondrial and peroxisomal altered energy metabolism as well as cytoskeletal derangement in RTD2 iPSCs and iPSC-derived motoneurons. So far, no attention has been dedicated to astrocytes.

**Results and discussion:**

Here, we demonstrate that *in vitro* differentiation of astrocytes, which guarantee trophic and metabolic support to neurons, from RTD2 iPSCs is not compromised. These cells do not exhibit evident morphological differences nor significant changes in the survival rate when compared to astrocytes derived from iPSCs of healthy individuals. These findings indicate that differently from what had previously been documented for neurons, RTD2 does not compromise the morpho-functional features of astrocytes.

## 1 Introduction

Riboflavin transporter deficiency (RTD), previously known as Brown-Vialetto-Van Laere syndrome (BVVLS, OMIM 613350, 614,707), is an autosomal recessive rare childhood neurodegenerative disease (<1/1,000,000 prevalence), characterized by sensorineural hearing loss, bulbar dysfunction, diffuse muscle weakness that may result in respiratory insufficiency. In particular, it can be caused by biallelic mutations in the SLC52A3 gene (613350) on chromosome 20p13 (OMIM 211530), encoding the Rf transporter RFVT3, mainly expressed in the testis, small intestine, is kidney, and placenta.

RTD Type 2 (RTD2, OMIM 614707), is caused by homozygous or compound heterozygous pathogenic variants in *SLC52A2* gene (607882) on chromosome 8q24 (Brown Charles Henry, 1894; Vialetto, 1936; Van Laere, 1966). *SLC52A2* encodes for the Rf transporter RFVT2, ubiquitously expressed in humans with the highest expression in the brain and spinal cord (Barile et al., 2013).

Since 2011, over 180 genetically confirmed RTD2 patients have been identified. Typical clinical features reported include optic atrophy, sensorineural hearing loss, muscle weakness, sensory ataxia, pontobulbar palsy, dysautonomia, and respiratory compromise [5–6, see Table 1]. Untreated patients with early onset generally rapidly decline, resulting in severe disability and early death due to respiratory failure (Green et al., 2010; Jaeger and Bosch, 2016). *SLC52A1* and *SLC52A3* genes, encoding Rf transporter RFVT1 and RFVT3, respectively, have also been linked to riboflavin-responsive disease. RFVT1/2/3 are transmembrane proteins (Fujimura et al., 2010), which mediate the absorption of Rf, as is not synthetized in humans but introduced with the diet (Suwannasom et al., 2020). Rf is the precursor of coenzymes obtained through enzymatic reactions: flavin mononucleotide (FMN) and flavin adenine dinucleotide (FAD). They take part in maintaining cellular redox balance: indeed, they are cofactors of several enzymes involved in both generating and scavenging of reactive oxygen species (ROS) and reactive nitrogen species (RNS) (Fransen et al., 2012). Excess of ROS and RNS can lead to oxidative stress and cause damages to biological molecules (DNA, RNA, proteins, lipids), thus impairing normal cell functions (Espinós et al., 2020). Various cell types are able to counteract ROS/RNS accumulation by activating a specific Nrf2-mediated antioxidant response and, in particular, astrocytes are more resistant than neurons because they can strongly upregulate Nrf2-mediated gene expression (Baxter and Hardingham, 2016).

**Table 1:** Clinical features associated with RTD2 (modified from https://curertd.org/what-is-rtd/symptoms/).

Symptom	RTD2 (Age: 0–5 years) *N* = 78	RTD2 (Age: 6–19 years) *N* = 16
Hearing Loss (Auditory Neuropathy)	29,4%	63%
Pontobulbar Palsy (Including feeding and speech issues)	9,1%	–
Muscle Weakness (Axonal neuropathy/mild demyelination)	29,4% (upper)	–
Sensory Gait Ataxia (Clumsy, staggering walk)	62,8%	32%
Vision Impairment (Optic atrophy with/without nystagmus)	27,4%	38%
Respiratory Compromise (Diaphragm weakness/sleep apnea)	12,1%	6%
Megalobastic Anemia	5%	–

Astrocytes are the most common glial cell type in the central nervous system (CNS) and are crucial for its proper function and health (Barres, 2008). They are multifunctional cells regulating neuronal synapses by promoting synaptogenesis and display morphological, functional, and molecular differences depending on the area in which they are located (John Lin et al., 2017). Additionally, astrocytes have a role in the uptake and metabolism of glutamate (Santello and Volterra, 2010), maintenance of blood–brain barrier (BBB) and modulation of extracellular space volume. Moreover, they supply energy substrates to neurons, release ROS scavengers and regulate the process of neurite outgrowth and myelination (Verkhratsky and Nedergaard, 2018).

Given the supporting role of astrocytes to neurons, changes in their behavior may greatly affect the pathogenesis of several neurodegenerative diseases. Indeed, recent studies have documented the contributing role of an altered function of these cells in amyotrophic lateral sclerosis (ALS) (Rossi and Cozzolino, 2021), Parkinson’s disease (PD) (Takahashi and Mashima, 2022), Alzheimer’s disease (AD) (Fakhoury, 2018; D’Ezio et al., 2021), leukodystrophies (Lanciotti et al., 2021), epilepsy (Binder and Steinhäuser, 2021) and HIV-associated neurocognitive disorders (HANDs) (Mastrantonio et al., 2019).

By using an *in vitro* disease model based on induced pluripotent stem cells (iPSCs) derived from RTD2 patients, we previously characterized the phenotype of RTD2 motoneurons focusing in particular on mitochondria, redox status, and neuronal morphology and activity (Rizzo et al., 2017; Marioli et al., 2020; Colasuonno et al., 2020b; Niceforo et al., 2021). Despite these insights, many aspects of the pathophysiological mechanisms implicated in RTD2 still deserve deep investigation, including those related to the cellular environment, astrocytes’ involvement and neuron–glia interactions. With this in mind, we used the patient-derived iPSC model to functionally profile patient-derived astrocytes to explore a possible role of these cells in the pathogenesis of RTD2 (Table 1).

## 2 Materials and methods

### 2.1 Derivation of iPSCs

The studies were conducted in compliance with the Code of Ethics of the World Medical Association (Declaration of Helsinki), and with national legislation and institutional guidelines (local institutional ethical committee, Ref. 1410_OPBG_2021, date of approval 11 February 2019). Informed consent was obtained from the subjects involved in the study. Patient skin fibroblasts were cultured in Dulbecco’s Modified Eagle Medium (DMEM D5671, Sigma Aldrich), supplemented with 10% of Fetal Bovine Serum (FBS, 10082–147, Gibco) and penicillin/streptomycin (15,140,148, Gibco) at 37°C, 5% CO_2_ and 21% O_2_. When the cells reached 80% of confluence, they were reprogrammed through nucleofection, as described in Okita et al. (2011). Using Epi5 Episomal iPSC Reprogramming Kit (A15960, Invitrogen) and the Nucleofection kit P2 solution (LOV4XP2024, Lonza) with 4D-Nucleofector System (Lonza), cells were plated in 6 well plates (3,516, Corning), pre-coated with Matrigel-Matrix hESC-qualified (354,277, Corning) in DMEM+FBS medium. After 24 h, the medium was changed with N2B27 medium (DMEM/F12 with L-Glutamine and 25 mM Hepes (ECM0095L, Euroclone), N-2 Supplement (17502–048, Gibco), B27 supplement (17504–044, Gibco), 100 μM of β-Mercaptoethanol (21985–023, Gibco), 0.1 mM MEM Non-Essential Amino Acids Solution (11140–050, Gibco). N2B27 medium was changed every day for 15 days and 100 ng/mL FGF2 (bFGF, 100–18B, Peprotech) was added fresh for each medium change. Then, Essential 8 medium (A1517001, Gibco) was used from the 15th until the 21st day of reprogramming. From day 21, cells were cultured in mTeSR1 Plus (05826, Stem Cell Technologies) until rounded and compact colonies appeared (about 30–40 days after the nucleofection). The colonies were picked under a sterile hood using the EVOS microscopy system (Thermofisher Scientific). The colonies were transferred to a 24 plate well pre-coated with Matrigel in mTeSR1 Plus medium and expanded for all experiments.

The generated iPSC lines carry the following *SLC52A2* pathogenic variants: c.155C > T (p.Ser52Phe) and c.935 T > C (p.Leu312Pro) (Patient 1, Pt1); c.505C > T (p.Arg169Cys) and c.1030_1031del (p.Leu344Alafs*100) (Patient 2, Pt2); c.505C > T (p.Arg169Cys) and c.593G > A (p.Trp198*) (Patient 3, Pt3). Pluripotency characterization of Pt1 iPSC lines had been previously reported (Niceforo et al., 2021). Control iPSCs (Ctrl iPSCs) used in this study were derived from healthy individuals derived from Coriell (AG28851 and AG28869) using non-integrating episomal technology. Images of control cells reported in figures represent representative images of one of the two control lines.

### 2.2 Clinical information

Clinical features of Pt1 had been previously reported (Niceforo et al., 2021). In particular, patient P1 developed macrocytic anemia and dysphagia at 3 months of age. Optic atrophy, axial muscle weakness, sensory ataxia, and respiratory compromise were noted at 1 year, and bilateral sensorineural hearing loss at 2 years. Patient P1 is now 13 years old and has remained neurologically stable since beginning riboflavin (75 mg/kg QID) and antioxidant therapy at 2.5 years of age. Moderate improvements in vision and hearing were measured during the first year after beginning riboflavin treatment, with the patient receiving a successful cochlear implant at age 3.9 years and currently wearing a night-time scoliosis brace since age 10.

Patient 2 is a girl presenting with rapid onset arm weakness and dysphagia at 8 months of age. At the time of diagnosis at 12 months old, severe optic atrophy and bilateral sensorineural deafness, sensory neuropathy, and complete diaphragm paralysis were noted. Genetic analysis performed by whole exome sequencing (WES) showed two mutations in *SLC52A2* (c.505C > T, p.Arg169Cys; c.1030_1031del, p.Leu344AlafsX100). Total flavin cerebrospinal fluid levels pre-Rf treatment were ~ 25% of controls, which normalized on 100 mg/kg of a 50/50 mix of riboflavin and riboflavin 5 phosphate QID. At age 6, the child continued to have severe upper limb and axial predominant muscle weakness, has developed severe scoliosis, and no significant improvement in vision or hearing. She continued to receive respiratory support but has regained partial diaphragm movement. This child recently developed cataracts requiring lens replacement, which has not previously been reported in children with RTD Type 2.

Patient 3 is a 4-year-old girl, third child born from healthy and non-consanguineous parents. Poor weight gain was reported at 2-month-old. At 6 months a psycho-motor development delay was evident. The baby showed marked and generalized hypotonia, had not achieved head control, and deep tendon reflexes were absent. Brain MRI showed a thinned appearance of the optic nerves. Visual evoked potential (VEP) was abnormal whereas auditory evoked potentials (AEP) did not detect brainstem abnormalities. ENG/EMG was conclusive for axonal sensorimotor neuropathy. Comprehensive metabolic screening including acyl-carnitines, plasma amino acids and urinary organic acids profiles were normal. Genetic analysis performed by WES showed two compound heterozygous pathogenic variants in *SLC52A2*, (c.505C > T, p.Arg169Cys; c.593G > A, p.Trp198*). Riboflavin was started at a dosage of 20 mg/kg/day. At the last clinical assessment (4 years), the baby presented poor growth, generalized hypotonia with kyphoscoliosis, nystagmus, possible visual pursuit, good head balance while maintained in a sitting position, independent sitting position with upper limbs support, and slight dysphagia mainly for liquids. She was orally fed by mouth with modified food consistency, and she had often respiratory infections. Polysomnography and video-fluoroscopy studies showed no abnormalities.

### 2.3 DNA sequencing

Ctrl and RTD2 iPSC pellets, obtained by centrifuging EDTA-detached cells (1,200 rpm, 5 min) were processed for genomic DNA extraction (740952.250, MACHEREY-NAGEL, Germany) following manufacturer’s instructions.

The coding sequences of exons 3 and 4 for patient 2, and exon 3 for patient 3 of the *SLC52A2* gene (NM_001363118) were amplified by PCR specific primers (available on request). Amplicons were purified using Exo-SAP (GE Healthcare) and directly sequenced using BigDye 3.1 chemistry (Applied Biosystems, Foster City, CA, United States) with an ABI Prism 3,130 xl automatic sequencer (Applied Biosystems).

### 2.4 Immunofluorescence assay for pluripotency

Cells were fixed in 4% paraformaldehyde (PFA, 157–8, Electron Microscopy Sciences) for 10 min at room temperature (RT) and washed twice in Dulbecco’s PBS w/o Calcium & Magnesium (PBS, ECB4004L, EuroClone). Cells were blocked with 5% bovine serum albumin (BSA, 10775835001, Roche) and permeabilized with 0.1% Triton X-100 (Sigma Aldrich) for 1 h, at RT. The primary antibodies used were: anti-OCT4 (1:100, rabbit; MA5-14845, Invitrogen), anti-SSEA4 (1:250, mouse; MA1-021, Invitrogen), anti-SOX2 (1:200, rat; 14–9,811-82, Invitrogen), anti-TRA-1-60 (1:100, mouse; SC-21705, Santacruz). They were incubated at 4°C overnight (ON). The secondary antibodies used were: AlexaFluor goat anti-mouse 488 (A11017, Invitrogen), AlexaFluor goat anti-mouse 555 (A21430, Invitrogen), AlexaFluor goat anti-rabbit 488 (A11070, Invitrogen), AlexaFluor anti-rat 488 (A11006, Invitrogen). They were diluted 1: 500 and incubated at RT for 1 h. The nuclei were counterstained with Hoechst 33342 (H3570, Thermofisher Scientific), diluted 1:10000 and incubated for 10 min at RT. Coverslips were mounted using 1:1 PBS:glycerol (G6279, Sigma Aldrich).

### 2.5 Alkaline phosphatase assay

Alkaline phosphatase (ALP, SCR004, Sigma Aldrich) staining was performed on cells fixed in 4% PFA. The samples were incubated at RT for at least 30 min with a solution based on naphthol AS-BI and fast red violet LB. The cells were photographed using a Leica DM1000 (Leica Microsystems) equipped with Leica LAS X software (Leica Microsystems).

### 2.6 *In vitro* trilineage differentiation assay

Trilineage differentiation was performed using the STEMdiff Trilineage Differentiation Kit (05230, STEMCELL Technologies). According to the protocol, iPSCs were plated on Matrigel with 10 μM Y-27632 (HY-10583, MedChem) and then treated with specific endoderm and mesoderm differentiation media for 5 days, or with ectoderm differentiation media for 7 days. Cells were fixed in 4% PFA, blocked with 5% BSA, and permeabilized with 0.1% Triton X-100 for 1 h, at RT, and then marked with: anti-SOX17 (1:3200, O/N at 4°C, rabbit, 81,778, Cell Signaling) to mark cells belonging to the endoderm; anti-Brachyury (1:1600, O/N at 4°C, rabbit, 81,694, Cell Signaling) for the identification of mesodermal cells; anti-NCAM (1:400, O/N at 4°C, rabbit, 89,861, Cell Signaling) to mark cells belonging to the ectoderm. AlexaFluor goat anti-rabbit 555 (A21430, Invitrogen) was used as secondary antibody and the nuclei counterstained with Hoechst 33342 (diluted 1:10000) and incubated for 10 min at RT. The coverslips were mounted using PBS:glycerol (1:1).

### 2.7 Maintenance of iPSCs

iPSCs were thawed in 6 well plates pre-coated with Matrigel-Matrix hESC-qualified (diluted 1:100) in DMEM, and cultured in mTeSR1 Plus, supplemented with 10 μM Y-27632. The proliferating iPSCs were kept in mTeSR1 Plus medium (37°C, 5% CO_2_ and 5% O_2_); when they reached 60–70% of confluence, they were split with ethylenediaminetetraacetic acid (EDTA, E1644, Sigma Aldrich) and expanded with mTeSR1 Plus or frozen in mFreSR (05855, Stem Cell Technologies).

### 2.8 Differentiation of iPSCs into astrocytes

iPSCs were differentiated into astrocytes (ASTROs) using an adapted protocol from Yan et al. (2013). According to this protocol, when the iPSCs reached the 80% of confluence, they were plated into 6well-plates pre-coated with Matrigel. The cells were split with Stempro Accutase (A1110501, Thermofisher Scientific) and plated at 2,5–3 × 10^5^ cells/well with Y-27632 to promote cell adhesion. After 24 h, the culture medium was switched to PSC Neural Induction Medium (A1647801, Gibco). The medium was changed every other day from day 0 to day 4; then, Neural Induction Medium was changed every day until day 7 when the cells reached the confluence. After the induction phase, primitive Neuronal Stem Cells (NSCs) were dissociated with Accutase and plated on Matrigel-coated 6well-plate at a density of 0.5–1 × 10^5^ cells per cm^2^ in an NSC expansion medium containing 50% Neurobasal medium, 50% DMEM/F12 (10,565,018, Thermofisher Scientific), and Neural Induction Supplement. During the expansion phase, the medium was changed every other day until day 7. When the 90% of confluence was reached, NSCs were split until step 7 and, in each passage, Y-27632 was added to the medium to prevent cell death. The NSCs can be cryopreserved in NSC expansion medium with 10% DMSO.

For the following 7 days, NSCs were cultured in DMEM/F-12 GlutaMAX supplemented with N-2 Supplement 1X, FBS 1% and 10 ng/mL bFGF. In the culture medium, 20 ng/mL recombinant human ciliary neurotrophic Factor (CNTF, 450–13, PeproTech) were added after 7 days from the beginning of ASTRO differentiation. At 90% confluence, ASTROs were split until passage 7 and, in each passage, the cells were cultured in the medium supplemented with Y-27632 to prevent adhesion difficulties. During each phase of differentiation, the cells were cultured at 37°C, 5% CO_2_ and 21% O_2_.

### 2.9 SDS-PAGE and western blotting

Preparation of cell extracts and western blot analyses were performed as follows: the cells were lysed in RIPA buffer (R0278, Sigma Aldrich) and supplemented with complete protease inhibitor cocktail (A32959, Sigma Aldrich). Proteins were separated by SDS-PAGE and following electrophoresis, proteins were transferred to nitrocellulose membrane using the Trans-Blot Turbo transfer system (Bio-Rad Laboratories). Membranes were blocked in 5% milk for 1 h, at RT. The primary antibodies used were: anti-GFAP 1:2000 (556,328, BD Pharmingen), anti-EAAT2 1:2000 (SC-365634, Santacruz), anti-GAPDH 1:10000 (G9545, Sigma Aldrich). They were blotted O/N at 4°C. The secondary antibodies were incubated for 1 h, at RT, and membranes stained with Clarity Western ECL Blotting Substrate (170–5,060, Biorad) and acquired using Uvitec. The analyses were performed using ImageJ Software and three biological replicates (n = 3 minimum) were performed for each experiment.

### 2.10 MTT assay

iPSC-derived astrocytes were cultured in 24 well Clear Flat Bottom plate (353,047, Falcon) at 75 × 10^3^ cells/well. After 48 h, cells were analyzed by MTT assay (3-(4,5-dimethylthiazol-2-yl)-2,5-diphenyltetrazolium bromide) as indicated by manufacturer’s instructions. MTT powder (M6494, Thermofisher Scientific) was diluted in H_2_O to obtain 0.5 mg/mL solution. The MTT solution was added to the cell with the final concentration of 10%. After 4 h of incubation at 37°C, MTT solvent (4 mM HCl, 0.1% NP40 in isopropanol) was added to permit the dissolution of formazan crystals and the cells were incubated for further 30 min. The optical density (O.D.) of each sample was measured at 570 nm using a microplate reader (BioTek ELx800 Absorbance Microplate Reader, Winooski). For each biological replicate (*n* ≥ 3), the data obtained from the RTD2 ASTROs were normalized to Ctrl ASTROs.

### 2.11 TUNEL assay

TdT-mediated dUTP-X nick end labeling (TUNEL, 11684795910, Roche) was performed to identify double-stranded and single-stranded DNA breaks, labeling the free 3′-OH terminus with modified nucleotides in an enzymatic reaction. The ASTROs were fixed with 4% PFA for 10 min at room temperature (RT); the samples were treated as described in the protocol and nuclei were counterstained using Hoechst 33342 diluted 1:10000 and incubated for 10 min at RT. Coverslips were mounted using PBS:glycerol (1:1). The ratio between the apoptotic and the total cells’ number was evaluated and GraphPad Prism was used to calculate the mean values ± SEM.

### 2.12 Differentiation of iPSCs into motoneurons

iPSCs were differentiated into motoneurons (MNs) using an adapted protocol from Corti et al. (2012). When the iPSCs reached 30–40% of confluence, they were cultured into NeuroCult NS-A Basal Medium Human medium (05750, Stem Cell Technologies) for 10 days. At the 10th day, the medium was supplemented with 0.1 μΜ retinoic acid (R2625, Sigma Aldrich). From the 17th to the 25th days, in addition to retinoic acid, 2 μΜ dorsomorphin (P5499, Sigma Aldrich) and 3 ng/mL activin A (120–14E, PeproTech) were added to the medium. From the 25th to the 30th days, the NeuroCult NS-A Basal Medium Human medium was replaced with BrainPhys Neuronal Medium (05790, Stem Cell Technologies) and the following factors were added: NeuroCult SM1 Neuronal Supplement (SM1, 05711, Stem Cell Technologies), N-2 Supplement, 200 μM ascorbic acid (A4403, Sigma Aldrich), 20 ng/mL recombinant human glial-derived neurotrophic factor (GDNF, 450–10, PeproTech), 20 ng/mL Recombinant human brain-derived neurotrophic factor (BDNF, 450–02, PeproTech). During differentiation, the cells were cultured at 37°C, 5% CO_2_ and 21% O_2_.

### 2.13 Neurite length assay

Motoneurons were plated at a density of 5,000 cells/well in a 96-well plate (92,696, TPP) pre-coated with Matrigel and neurites’ length was measured at the end of differentiation (30 days) using the live imaging system Incucyte SX5 (Sartorius). Phase-contrast images were acquired and processed using NeuroTrack Incucyte software. The parameters were optimized according to the workflow outlined in the manufacturer’s manual. Raw data neurite lengths were exported to Microsoft Excel and GraphPad Prism was used to obtain the graph, calculate mean values ± SEM and perform *ad hoc* statistical analyses.

### 2.14 Immunostaining analyses

For immunochemistry, cells were fixed with 4% PFA for 10 min at room temperature (RT) and treated with a permeabilizing and blocking solution containing PBS, 5% BSA and 0.1% Triton X-100 for 1 h. The primary antibodies used for these studies were: anti-NESTIN, 1:300 for O/N at 4°C (MAB5326, Millipore); anti-PAX6, 1:100 for O/N at 4°C (SC-32766, Santacruz); anti-GFAP, 1:50 for ON at 4°C (Z0334, DAKO); anti-GFAP, 1:50 for 1 h at 37°C (556,328, Pharmingen); anti-TUJ1, 1:500 for 2 h at RT (T2200, Sigma). The secondary antibodies used were: AlexaFluor goat anti-mouse 488, AlexaFluor goat anti-mouse 555, AlexaFluor goat anti-rabbit 488, AlexaFluor goat anti-rabbit 555. They were diluted 1: 500 and incubated at RT for 1 h. Nuclei were counterstained using Hoechst 33342 (diluted 1:10,000) and incubated for 10 min at RT, and coverslips were mounted using PBS:glycerol (1:1). Three independent experiments were performed for each sample. All images were acquired using the Olympus Fluoview FV3000 Confocal Laser Scanning Microscope (Evident-Olympus, Tokyo, Japan). The microscope was equipped with a main laser combiner source (405 nm, 488 nm, 561 nm, 640 nm), a sub laser combiner (514 nm, 594 nm) and 3 Internal Spectral Detector Channels (PMT) and 2 Cooled GaAsP photomultiplier. Sequential confocal images were acquired using 40X/1.40 N.A. or 60X/1.42 N.A. objectives with a 1,024 × 1,024 format, scan speed 8us/pixel, and z-step size of 0.4 μm. Acquisition settings (i.e., lasers’ power, beam splitters, filter settings, pinhole diameters and scan mode) were the same for all examined samples of each staining.

### 2.15 RNA isolation and reverse transcriptase-polymerase chain reaction analyses

RNA was extracted from Ctrl and RTD2 iPSCs, iPSCs-derived MNs and ASTROs using Trizol Reagent (15,596,026, Ambion by Life Technologies), according to the manufacturer’s instructions. Each RNA sample was quantified using the Nanodrop 2000 Spectrophotometer (Thermo Fisher Scientific) and 1 μg of total RNA was reverse transcribed using LunaScript RT SuperMix Kit (E3010, BioLabs) following the instructions. Three independent experiments were performed for each sample.

### 2.16 Quantitative real-time PCR

Quantitative Real-Time PCR (qRT-PCR) experiments were performed using Fast SYBR Green Master Mix (4,385,612, Applied Biosystems) and the QuantStudio 7 Pro Real-Time PCR System (Applied Biosystems). The analyses were performed following the ΔΔCt method: the data were represented as fold increase versus control sample, calculated by the 2^[−(ΔΔC)] formula. Expression levels of different genes were compared with those of the housekeeping gene GAPDH, and they were normalized to control’s expression. The values were expressed in arbitrary units. Three independent Quantitative Real-Time PCR were performed for each sample. Below is a table (Table 2) with the sequence of all primers used.

**Table 2:** Table reporting the primer sequences used for the Quantitative Real-Time PCR experiments.

Human gene	Primer (5′--3′) Sense antisense
Glyceraldehyde 3-phosphate dehydrogenase (GAPDH)	Forward 5′-TTGTTGCCATCAATGACCC-3′ Reverse 5′-CTTCCCGTTCTCAGCCTTG-3′
Glutathione peroxidase (GPX3)	Forward 5′-CATTCGGTCTGGTCATTCTG-3′ Reverse 5′-CCTGGTCGGACATACTTGA-3′
Heme oxygenase (HO-1)	Forward 5′-CGGGCCAGCAACAAAGTG-3′ Reverse 5′-AGTGTAAGGACCCATCGGAGAA-3′
Octamer-binding transcription factor 4 (OCT4)	Forward 5′-AGCGAACCAGTATCGAGAAC-3′ Reverse 5′-TTACAGAACCACACTCGGAC-3′
Superoxide dismutase 1 (SOD1)	Forward 5′- AGTAATGGACGAGTGAAGG-3′ Reverse 5′- GGATAGAGGATTAAAGTGAGGA-3′
Superoxide dismutase 2 (SOD2)	Forward 5′-AATGGTGGTGGTCATATCA-3′ Reverse 5′-CCCGTTCCTTATTGAAACC-3′
System X_c_^−^ (xCT)	Forward 5′-GGTGGTGTGTTTGCTGTC-3′ Reverse 5′-GCTGGTAGAGGAGTGTGC-3′
Nuclear factor erythroid 2–related factor 2 (NRF2)	Forward 5′-ATGCCCTCACCTGCTACTTT-3′ Reverse 5′-AGTGAAATGCCGGAGTCAG-3′
SRY (sex determining region Y)-box 2 (SOX2)	Forward 5’-AGCTACAGCATGATGCAGGA-3’ Reverse 5’-GGTTCATGGAGTTGTACTGCA-3’
Kruppel-like factor 4 (KLF4)	Forward 5’-CATCTCAAGGCACACCTGCGAA-3’ Reverse 5’-TCGGTCGCATTTTTGGCACTGG-3’

To assess the mRNA levels of *SLC52A2*, qPCR experiments were performed using TaqMan Fast Universal PCR Master Mix (4,352,042, Thermofisher Scientific) and the following primers: *SLC52A2* (4,331,182, Hs00364295_m1, Thermofisher Scientific) and *GAPDH* (4,331,182, Hs99999905_m1, Thermofisher Scientific). The analyses were performed as described above.

### 2.17 Statistical analyses

The data obtained were represented using mean ± SEM. For all experiments, multiple technical replicates and biological replicates were utilized and a minimum of three independent experiments were performed for each assay. Quantification and statistical analyses of the control cells were reported as the mean of the data derived from the two cell lines (AG28851 and AG28869). Significance was tested using ordinary one-way ANOVA (parametric tests) for normally-distributed data. GraphPad-Prism software (Prism 8.0.2, GraphPad Software) was used to analyze the data.

## 3 Results

### 3.1 Characterization of two new patient-derived iPSCs and RTD2 motoneurons

**Figure 1:**
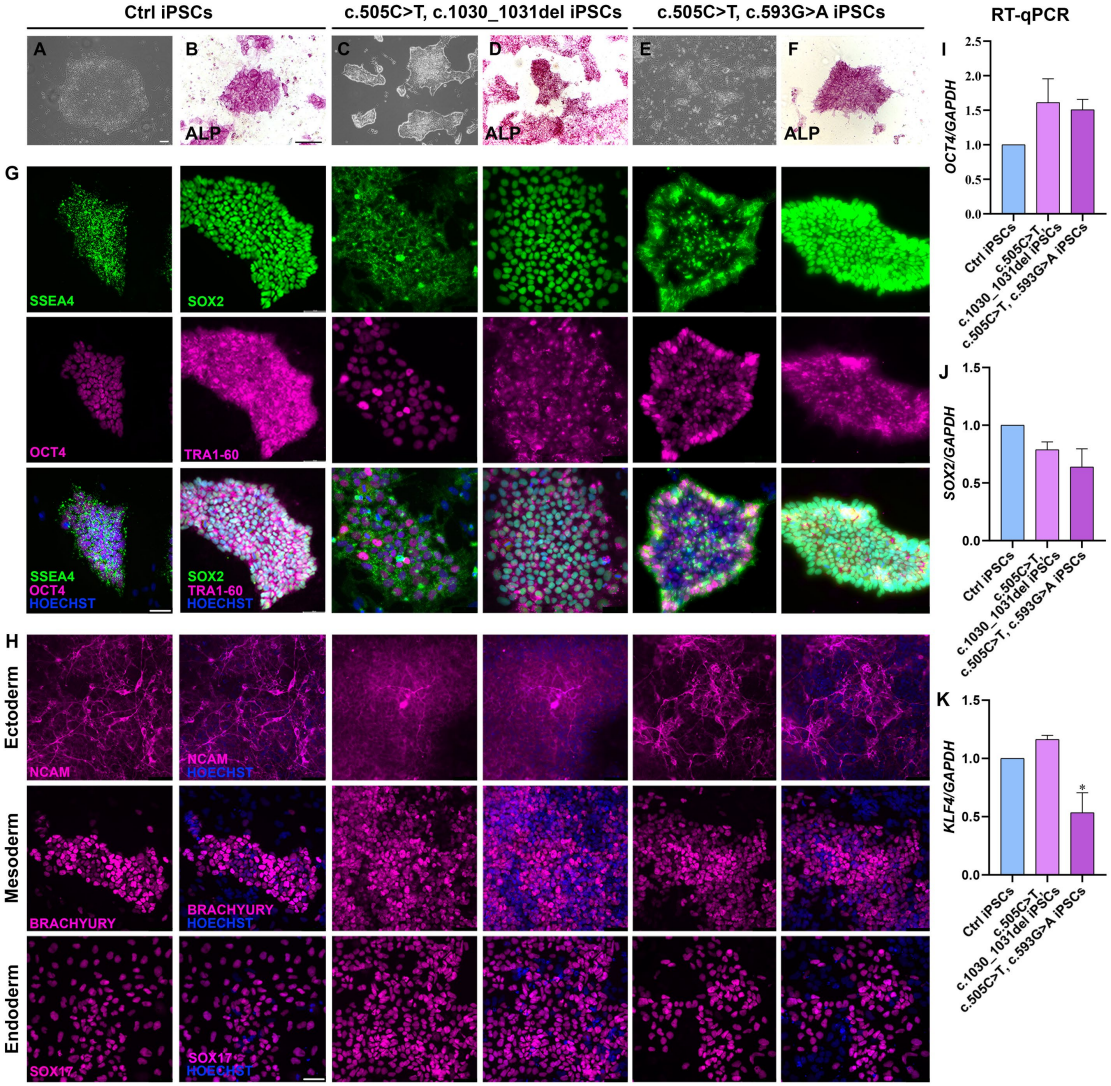
Characterization of iPSCs obtained from RTD2 patients with mutations in *SLC52A2*. **(A,C,E)** Brightfield images of Ctrl and RTD2 patient-derived iPSCs (obtained from an optical microscope with 4x magnification), showing the rounded shape of the obtained colonies (scale bar = 20 μm). (**B,D,F)** ALP staining showing iPSC positivity to alkaline phosphatase (scale bar = 200 μm). **G)** Immunofluorescence assays for the pluripotency markers SSEA4 and SOX2 (in green), OCT4 and TRA1-60 (in magenta). Nuclei are counterstained with Hoechst (in blue) (scale bar = 50 μm). **(H)** Characterization of the ability of Ctrl and RTD2 iPSCs to differentiate into the three germ layers. The images show that both Ctrl and RTD2 iPSCs are able to differentiate in cells belonging to the ectoderm (NCAM, in magenta), mesoderm (BRACHIURY, in magenta) and endoderm (SOX17, in magenta) lineages. Nuclei are counterstained with Hoechst (in blue) (scale bar = 50 μm). The mRNA expression levels of **(I)** *OCT4* [*F* (2, 9) = 2.268, *p* = 0.1594, ANOVA], **(J)** SOX2 [*F* (2, 9) = 3.385, *p* = 0,0801, ANOVA] and **(K)** KLF4 [*F* (2, 7) = 12.39, *p* = 0.0050, ANOVA] evaluated by RT-qPCR, using GAPDH as housekeeping gene. Data are normalized to Ctrl and are presented as mean ± SEM of three independent biological replicates (*n* = 3). *p ≤ 0.05, according to multiple comparisons of Ctrl iPSCs vs. RTD2 iPSCs (ordinary one-way ANOVA).

Two lines of RTD2 patients’ fibroblasts with biallelic pathogenic variants in *SLC52A2*, obtained from skin biopsies, were reprogrammed into iPSCs introducing episomal vectors by nucleoporation. Four clones from each primary fibroblast line were selected based on the ESC-like morphology (rounded shape colonies with defined borders) and picked for following amplification and characterization (Figures 1A,C,E). Sanger sequencing confirmed that the *SLC52A2* mutations identified in the patients’ fibroblasts were present following reprogramming (Supplementary Figures S1A,B). Pluripotency of clones was confirmed by enzymatic assay using ALP (Figures 1B,D,F) and by Real-time PCR (qRT-PCR) evaluating pluripotency markers, in particular *OCT4* (Figure 1I), *SOX2* (Figure 1J), and *KLF4* (Figure 1K).

**Figure 2:**
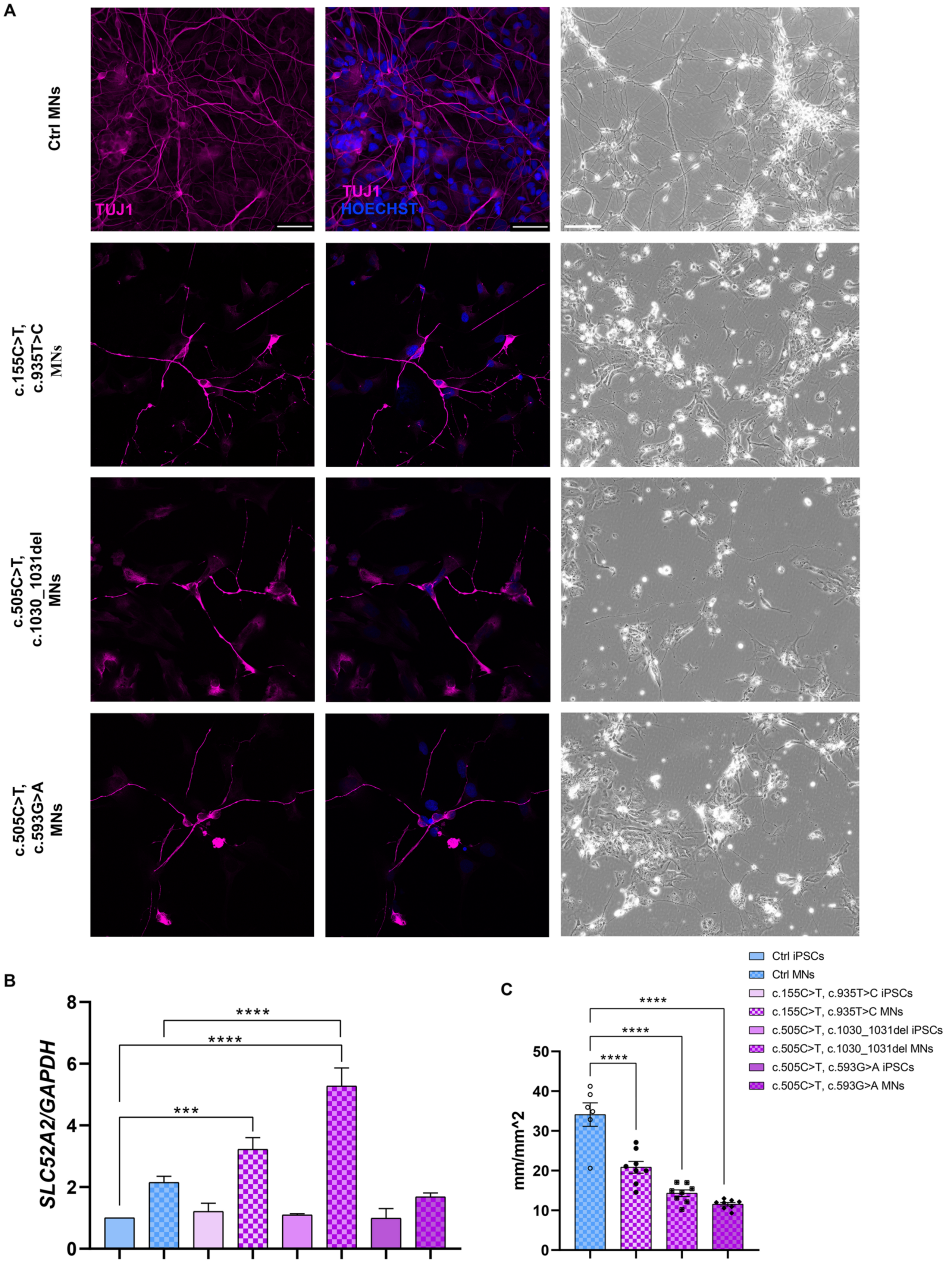
Characterization of Ctrl and RTD2 MNs. **(A)** Bright field and immunofluorescence images of TUJ1/β III tubulin (in magenta) show shorter neurites in RTD2 MNs compared to control cells. Nuclei are counterstained with Hoechst (in blue). Scale bars = 50 μm. **(B)** qRT-PCR analyses of *SLC52A2* genes in iPSCs and iPSC-derived MNs [*F* (7, 12) = 28,56, *p* < 0,0001, ANOVA], using *GAPDH* as housekeeping gene. **(C)** The mean of neurites’ length at day 30 of differentiation was measured using the NeuroTrack Incucyte software [*F* (3, 26) = 40,31, *p* < 0,0001]. Data are presented as mean ± SEM, obtained from three biological replicates (*n* = 3). *** *p* ≤ 0.001, **** *p* ≤ 0.0001, according to multiple comparisons of Ctrl iPSCs vs. RTD2 iPSCs or Ctrl and RTD2 MNs (ordinary one-way ANOVA).

The pluripotency of the picked clones was further confirmed with immunofluorescence assays using the pluripotency markers OCT4, SOX2, SSEA4 and TRA1-60 (Figure 1G). In addition, we tested their ability to differentiate into lineages belonging to the three germ layers (endoderm, mesoderm, and ectoderm) by Trilineage differentiation assay. Immunofluorescence assays were used to assess the positivity of the obtained iPSC clones to the following specific markers: SOX17 for the endoderm; TBXT or BRACHIURY for the mesoderm; and NCAM for the ectoderm (Figure 1H). Furthermore, we differentiated Ctrl and RTD2 iPSCs into motoneurons as described in Corti et al. (2012) to analyze the differentiating ability of the iPSCs derived from the two newly obtained patients’ lines. Immunofluorescence of TUJ1 confirmed that their neuronal morphology is compromised (Figure 2), as already demonstrated for neuronal cultures derived from patient 1 and other RTD2 patients (Marioli et al., 2020; Colasuonno et al., 2020a; Niceforo et al., 2021). Motoneurons derived from the previously established and newly generated RTD2 iPSC lines showed significantly shorter neurites than Ctrl motoneurons (Figures 2A,C). We evaluated the *SLC52A2* mRNA levels in Ctrl and RTD2 iPSCs and Ctrl motoneurons showed increased levels versus Ctrl iPSCs, and interestingly, increased *SLC52A2* levels were found in RTD2 motoneurons derived from patient 1 (c.155C > T; c.935 T > C) and patient 2 (c.505C < T; c.1030_1031del) compared to Ctrl motoneurons (Figure 2B).

### 3.2 The generation of ctrl and RTD2 astrocytes

**Figure 3:**
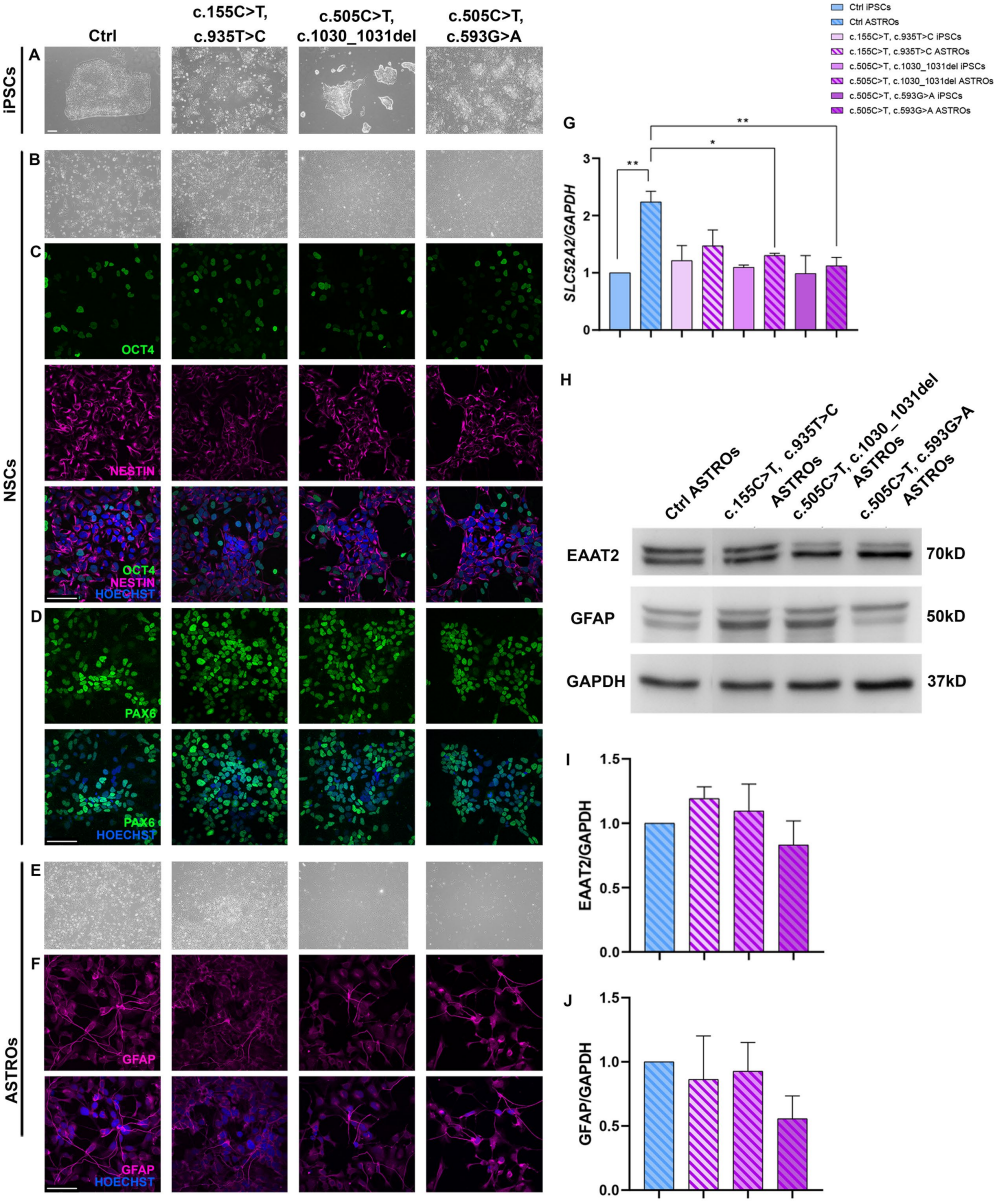
Characterization of Ctrl and RTD2 NSCs and ASTROs. **(A)** Brightfield images of Ctrl and RTD2 patient-derived iPSCs. **(B)** Brightfield and immunofluorescence images of Ctrl and RTD2 NSCs, highlighting the positivity to **(C)** NESTIN (in magenta), a marker of neuronal progenitors and OCT4 (in green), a marker of pluripotent cells. **(D)** Immunofluorescence analyses of PAX6 (in green), transient maker of neural progenitor cells. Nuclei are counterstained with Hoechst (in blue) (scale bar = 25 μm). **(E)** Brightfield images of Ctrl and RTD2 ASTROs (obtained from an optical microscope with 4x magnification). **(F)** Images of immunofluorescence assays for GFAP staining astrocytes (in magenta). **(G)** *SLC52A2* mRNA levels [*F* (7, 16) = 4.4, *p* = 0.0063, ANOVA] evaluated by RT-qPCR, using *GAPDH* as housekeeping gene. Data are normalized to Ctrl iPSCs and are presented as mean ± SEM of three independent biological replicates (*n* = 3). * *p* ≤ 0.05, ** *p* ≤ 0.01, according to multiple comparisons (ordinary one-way ANOVA). **(H)** Western blot analyses showing the expression levels of GFAP and EAAT2. GAPDH was used as internal control. The experiments were performed on protein extracts of Ctrl and RTD2 ASTROs after 60 days of differentiation. **(I)** Bar graphs show the quantification of EAAT2 [*F* (4, 10) = 1.286, *p* = 0.3389, ANOVA] relative to GAPDH levels. **(J)** Bar charts show the quantification of GFAP [*F* (4, 10) = 1.830, *p* = 0.1998, ANOVA] relative to GAPDH levels. Data are the mean fold induction with respect to Ctrl ASTROs and are presented as mean ± SEM of three independent biological replicates (*n* = 3).

To investigate the role of astrocytes in RTD2 pathology, Ctrl and RTD2 iPSCs (Figure 3A) were differentiated into astrocytes in a 60-day protocol. NSCs were derived from RTD2 and Ctrl iPSCs following 30 days of differentiation (Figure 3B) using an adapted protocol from Yan et al. (2013) and immunofluorescence analyses were performed with NESTIN and PAX6, as markers of neuronal progenitor cells (Figures 3C,D). The cellular morphology changed from a rounded shape to an elongated form and the percentage of OCT4 positive cells (representing the pluripotent cells) correctly decreased following differentiation in Ctrl and RTD2 cells (Figure 3C).

Once the NSCs were obtained, cells were differentiated for other 30 days to obtain mature astrocytes (Figure 3E). To demonstrate that iPSCs successfully differentiated into astrocytes, immunofluorescence and western blotting analyses for the astrocytic marker GFAP were performed (Figures 3F,H). Further western blotting analyses confirmed the protein expression of another astrocytic marker, EAAT2, in the obtained astrocyte cultures (Figure 3H). Statistical differences were not observed between Ctrl and RTD2 astrocytes, both in GFAP (Figure 3J) and EAAT2 (Figure 3I) levels, indicating that RTD2 iPSCs are able to undergo astrocyte differentiation similarly to Ctrl iPSCs.

The expression level of *SLC52A2* mRNA was evaluated using qRT-PCR analyses. The results showed that Ctrl astrocytes had significantly higher levels compared to Ctrl iPSCs. Similarly, RTD2 astrocytes had higher levels compared to RTD2 iPSCs (Figure 3G). However, there was no significant statistical difference observed among Ctrl and RTD2 iPSCs (Figure 3G).

### 3.3 Cell viability and death studies of ctrl and RTD2 astrocytes

**Figure 4:**
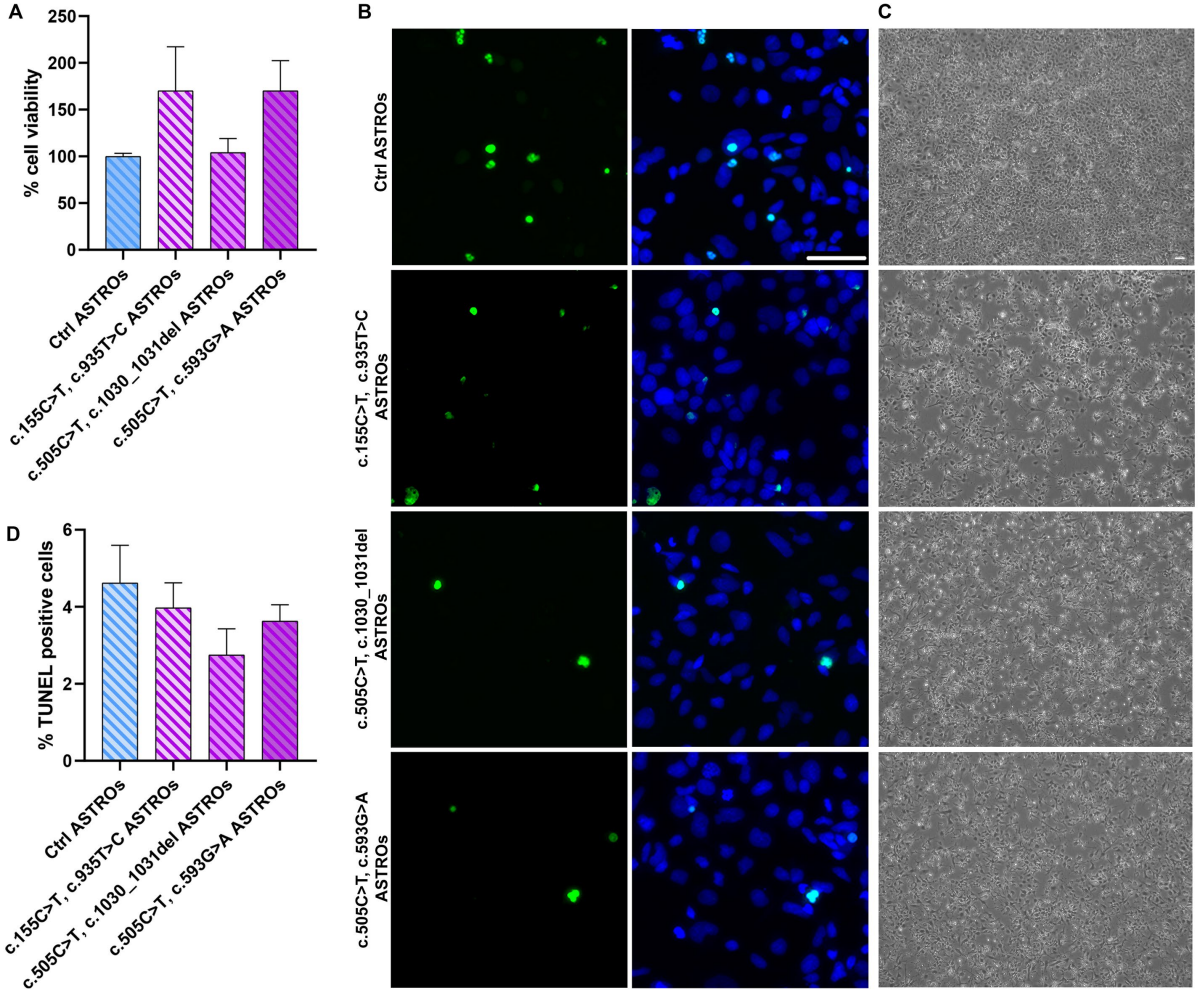
Analyses of viability and cell death in RTD2 and Ctrl ASTROs. **(A)** Bar graph depicting cell viability using MTT assay [*F* (4, 34) = 1.764, *p* = 0.1590, ANOVA]. Data are normalized to Ctrl ASTROs and presented as the mean ± SEM of three biological replicates (*n* = 3). **(B)** Confocal micrographs of astrocytes cultures following TUNEL assay (in green) and counterstaining of nuclei with Hoechst (in blue) (scale bar = 50 μm). **(C)** Bright field images of Ctrl and RTD2 iPSC-derived astrocytes (scale bar = 25 μm). **(D)** Bar graph depicting the quantification of TUNEL positive cells [*F* (4, 70) = 1.258, *p* = 0.2948, ANOVA] over the total number of cells. Data are presented as the mean ± SEM of three biological replicates (*n* = 3). The total number of analyzed cells is ~5,600 cells.

To evaluate the viability of RTD2 astrocytes compared to Ctrl ones, we measured their cell viability using the MTT assay. The results showed no significant difference between Ctrl and RTD2 astrocytes (Figure 4A). Furthermore, the TUNEL assay was used to analyze cell death in Ctrl and RTD2 astrocytes and the results confirmed no differences between the two groups. The quantification of the dead cells over the total number of cells (Figure 4B) showed that the percentage of TUNEL–positive cells was similar in Ctrl and RTD2 astrocytes (Figure 4C).

### 3.4 Analyses of antioxidant response in iPSCs, motoneurons and astrocytes using qRT-PCR

**Figure 5:**
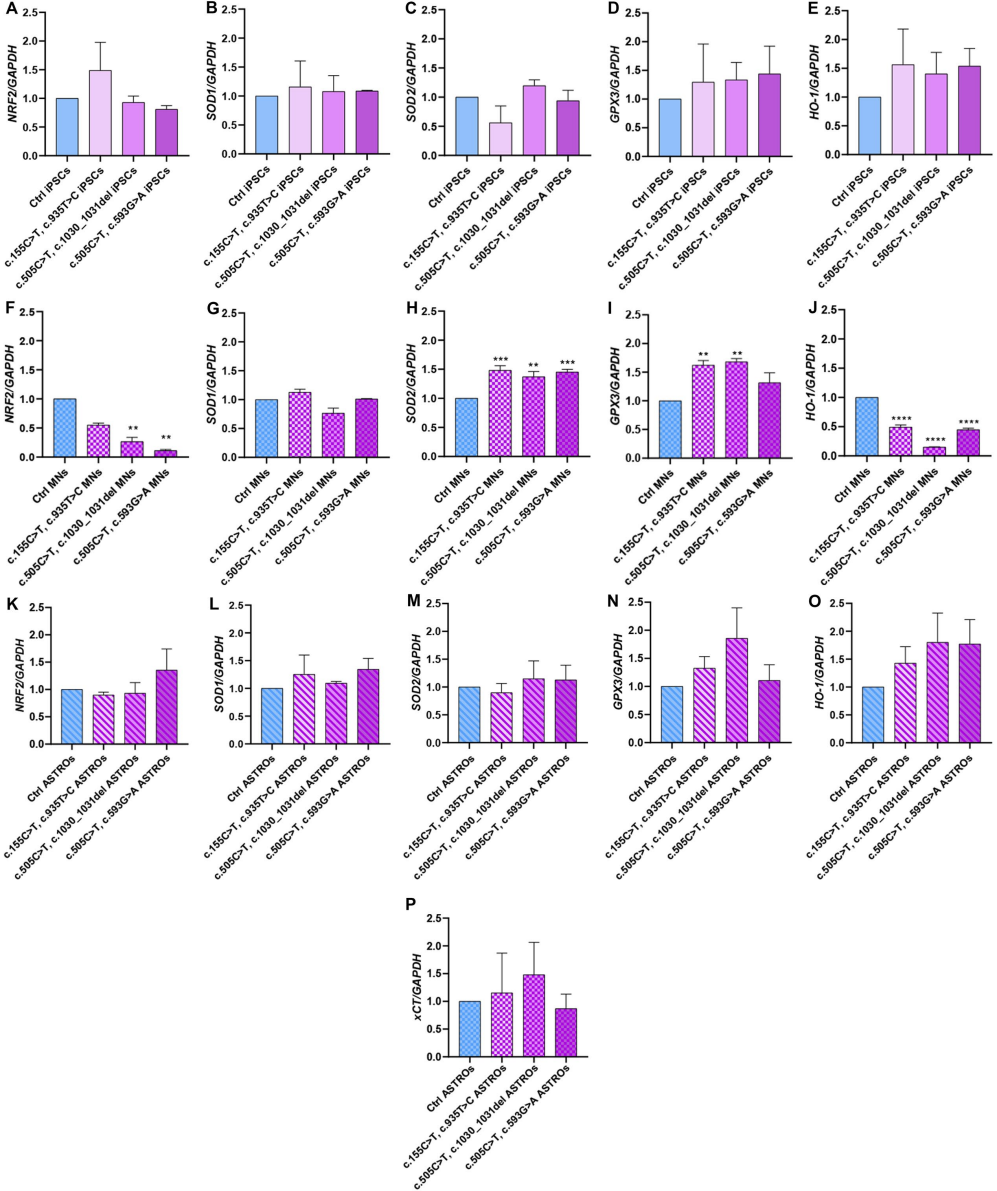
qRT-PCR analyses of ARE genes involved in the regulation of the antioxidant response in iPSCs, iPSC-derived MNs and ASTROs. The mRNA levels were evaluated for: *NRF2* (Nuclear factor erythroid 2-related factor 2) in **(A)** iPSCs [*F* (4, 17) = 7.162, *p* = 0.0014, ANOVA], **(F)** MNs [*F* (4, 10) = 8.295, *p* = 0.0032, ANOVA] and **(K)** ASTROs [*F* (4, 14) = 0.7346, *p* = 0.5835, ANOVA]; *SOD1* (Superoxide dismutase 1) in **(B)** iPSCs [*F* (4, 5) = 1.001, *p* = 0.4854, ANOVA], **(G)** MNs [*F* (4, 10) = 7.324, *p* = 0.0050, ANOVA] and **(L)** ASTROs [*F* (4, 10) = 1.462, *p* = 0.2846, ANOVA]; *SOD2* (Superoxide dismutase 2) in **(C)** iPSCs [*F* (4, 10) = 1.264, *p* = 0.3463, ANOVA], **(H)** MNs [*F* (4, 10) = 75.12, *p* < 0,0001, ANOVA] and **(M)** ASTROs [*F* (4, 10) = 4.232, *p* = 0.0293, ANOVA]; *GPX3* (Glutathione peroxidase 3) in **(D)** iPSCs [*F* (4, 10) = 1.038, *p* = 0.4344, ANOVA], **(I)** MNs [*F* (4, 10) = 57.65, *p* < 0,0001, ANOVA] and **(N)** ASTROs [*F* (4, 10) = 5.497, *p* = 0.0133, ANOVA]; *HO-1* (heme oxygenase 1) in **(E)** iPSCs [*F* (4, 10) = 1.015, *p* = 0.4446, ANOVA], **(J)** MNs [*F* (4, 10) = 251.5, *p* < 0,0001, ANOVA] and **(O)** ASTROs [*F* (4, 10) = 4.300, *p* = 0.0280, ANOVA]; *xCT^−^* (Cystine/glutamate transporter) in **(P)** ASTROs [*F* (4, 10) = 5.857, *p* = 0.0108, ANOVA]. Data are normalized to Ctrl and are presented as mean ± SEM of three independent biological replicates (*n* = 3). Following multiple comparisons according to ordinary one-way ANOVA: *p ≤ 0.05; **p ≤ 0.01; ***p ≤ 0.001; ****p ≤ 0.0001 between Ctrl and RTD2 iPSCs or Ctrl and RTD2 MNs or Ctrl and RTD2 ASTROs.

Given the pivotal role of astrocytes in supporting neuronal function and since RTD2 motoneurons and iPSCs were documented to display energy dysmetabolism and redox imbalance (Colasuonno et al., 2020a,b), we investigated these features analyzing the molecular mechanisms underlying the cellular redox homeostasis in Ctrl and RTD2 iPSCs, motoneurons and astrocytes. To this aim, we performed qRT-PCR analyses to evaluate the expression of genes involved in the antioxidant response. Firstly, we analyzed the expression of *NRF2*, since it is the major orchestrator of the cellular antioxidant defense. The results indicated that in RTD2 iPSCs, *NRF2* expression was comparable with that of Ctrl iPSCs (Figure 5A). *NRF2* is a transcription factor that regulates several phase II detoxifying and antioxidant enzymes containing the antioxidant responsive element (ARE) in the promoter region, such as *SOD1, SOD2, GPX3, HO-1*, and *System X_C_^−^* (xCT subunit), thus we investigated the expression level of these *ARE* genes in RTD2 and Ctrl cells. The data obtained from the qRT-PCR analysis showed that the expression level of *SOD1* (Figure 5B), *SOD2* (Figure 5C), *GPX3* (Figure 5D) and *HO-1* (Figure 5E) were comparable between Ctrl and RTD2 iPSCs.

After differentiation from iPSCs, we analyzed the same genes in motoneurons. We observed a reduction in *NRF2* expression levels in all RTD2 motoneurons compared to Ctrl motoneurons. Specifically, there was a statistically significant reduction in motoneurons generated from Pt1, Pt2 and Pt3 (Figure 5F). In relation to the expression of ARE genes, it was observed that the *SOD1* mRNA levels in RTD2 motoneurons for all patients were similar to those in Ctrl motoneurons (Figure 5G). Notably, an altered expression of *SOD2* was observed in RTD2 motoneurons, with a significant increase in all RTD2 motoneurons (Figure 5H) compared to Ctrl motoneurons. Similarly, *GPX3* expression levels were increased in all RTD2 motoneurons (Figure 5I). Regarding the mRNA levels of *HO-1*, a statistically significant reduction was observed in all RTD2 motoneurons compared to Ctrl motoneurons (see Figure 5J).

Concerning the analyses on astrocytes, the expression levels of *NRF2* were found to be similar between Ctrl and RTD2 astrocytes (Figure 5K). Similarly, no significant differences in the mRNA levels of *SOD1* (Figure 5L), *SOD2* (Figure 5M), *GPX3* (Figure 5N), *HO-1* (Figure 5O) and *xCT* (Figure 5P) among lines were observed.

## 4 Discussion

RTD2 is a rare neurodegenerative disorder caused by mutations in *SLC52A2* gene, encoding the Rf transporters RFVT2. Riboflavin, known as vitamin B2, is a water-soluble vitamin that cannot be produced endogenously by humans and is an indispensable part of the diet. Noteworthy, this vitamin is the precursor of the FMN and FAD coenzymes. Since RTD2 is rare and, as other neurodegenerative diseases, the *in vivo* models fail to recapitulate the human clinical phenotype and disease progression (Colasuonno et al., 2022), hiPSCs represent an informative model system to explore the mechanism of disease and pathophysiology.

It is known that RTD2 motoneurons show morphological/cytoskeletal abnormalities and activity, and mitochondrial alterations (Colasuonno et al., 2020a,b; Niceforo et al., 2021), which are partially recovered after the treatment with a combination of Rf and antioxidants (Marioli et al., 2020). Despite this, unfortunately, not all patients are responsive to Rf, as some patients do not benefit from Rf supplementation or only for a limited amount of time. With the aim to more deeply understand the RTD2 pathophysiology, we investigated the involvement of astrocytes in RTD2 pathology, based on the important role of these cells in neuronal function (Dermitzakis and Clark, 2002; Odom et al., 2007; Ilieva et al., 2009; Ciccolella et al., 2012). We, therefore, focused our attention on a possible contribution of astrocytes to RTD2 pathogenesis, in particular by analyzing the efficiency of astrocyte differentiation from patient-derived iPSCs and their redox status.

We successfully differentiated RTD2 iPSCs into astrocytes. Firstly, we differentiated Ctrl and RTD2 iPSCs into NSCs expressing the neuronal progenitors’ markers PAX and NESTIN (Figure 3). Then, to evaluate the efficiency of astrocyte differentiation, we performed immunostaining for GFAP and western blotting for GFAP and EAAT2, both markers of mature astrocytes.

The positivity of both Ctrl and RTD2 cells (Figure 3) confirms that RTD2 iPSCs can successfully differentiate into astrocytes (Su et al., 2003; Fox et al., 2004). These findings suggest that the difficulties observed in RTD2 iPSCs differentiating into motoneurons (Figure 2A) do not apply when differentiating into astrocytes (Figure 3).

We evaluated the expression level of *SLC52A2* mRNA. By qRT-PCR, we observed no differences in *SLC52A2* expression between Ctrl and RTD2 iPSCs. Noteworthy, RTD2 astrocytes showed reduced *SLC52A2* mRNA levels when compared to Ctrl astrocytes, but further specific studies will be necessary to investigate this peculiar feature of RTD2 astrocytes. Interestingly, *SLC52A2* mRNA expression was found to be higher in RTD2 motoneurons compared to Ctrl motoneurons (Figure 2B). This suggests that motoneurons require RFVT2 to perform their functions, and in RTD2 neurons where the RFVT2 protein is partially functional, the cell transcriptional machinery is forced to translate more mRNA copies of the *SLC52A2* gene as a compensatory mechanism.

To accomplish a better characterization of RTD2 astrocytes, we evaluated their viability using MTT and TUNEL assays, demonstrating no significant changes in both these features in Ctrl versus RTD2 astrocytes, underling that *SLC52A2* mutations did not influence these features in RTD2 astrocytes (Figure 4).

In RTD2 pathology, mutations of the Rf transporters induce most likely alterations in the absorption of Rf, consequently causing defects in the levels of FAD and FMN, and affecting all molecular mechanisms dependent on them, and particularly the redox status regulation (Barile et al., 2016). For this reason, we decided to analyze the mRNA levels of ARE genes involved in the antioxidant response of RTD2 iPSCs, motoneurons and astrocytes. We first evaluated *NRF2* mRNA expression, as NRF2 is a key orchestrator of the antioxidant response required to counteract ROS accumulation (Ma, 2013). RT-qPCR analyses indicated that *NRF2* levels were the same in both Ctrl and RTD2 iPSCs (Figure 5A). Although the oxidative processes increase following neuronal differentiation, as the energy production in neurons relies on oxidative phosphorylation (Trigo et al., 2022), *NRF2* levels in RTD2 motoneurons were reduced with respect to Ctrl motoneurons. The obtained results indicate that this antioxidant defense pathway is altered in RTD2 motoneurons (Figure 5F), and this is consistent with our previously reported data (Niceforo et al., 2021). NRF2-mediated antioxidant response is particularly active in astrocytes. Indeed, several studies demonstrated the role of astrocytic NRF2 signaling in neuroprotection (Tanaka et al., 1999) as well as in neurotoxicity (D’Ezio et al., 2021). In an *in vitro* model of Alzheimer’s disease, the activation of NRF2-mediated antioxidant response and the consequent upregulation of system Xc^−^ is involved in neuronal death due to increased glutamate release (D’Ezio et al., 2021). Therefore, we hypothesized that the behavior of astrocytes may be relevant in the pathogenesis of RTD2, although the most obvious effects of the disease are on motoneurons. Our findings indicate that *NRF2* levels were the same in both RTD2 and Ctrl astrocytes (Figure 5K) and are consistent with an unmodified phenotype of RTD2 astrocytes, and in line with the absence of morphological differences with respect to Ctrl astrocytes.

We also evaluated the expression of some NRF2-driven ARE genes as *SOD1, SOD2, GPX3, HO-1* and *xCT* in both Ctrl and RTD2 iPSCs, motoneurons and astrocytes (Figure 5). Interestingly, we found that the expression of all these genes was similar in Ctrl and RTD2 iPSCs, thus confirming the absence of impairment of the antioxidant response in RTD2 iPSCs.

In RTD2 motoneurons, *NRF2* levels were reduced in RTD2 compared to Ctrl motoneurons. In RTD2 cells, we also observed higher mRNA levels of some ARE genes when compared to the control cells. In this respect, it should be recalled that in addition to transcriptional control, epigenetic regulation, and post-transcriptional modifications can also play essential roles in controlling the levels of these phase-II enzymes. These processes include, for example, stabilizing *SOD* mRNAs and regulating their translation (Miao and St Clair, 2009). In all RTD2 motoneurons, we observed that the levels of *SOD2* mRNA were increased with respect to Ctrl motoneurons (Figure 5H), suggesting that patients motoneurons try to counteract oxidative stress through different ROS-scavenging systems. Among ROS species, O_2_^−^ is one of the most harmful to cell health. For this reason, enzymes have evolved to metabolize it as SOD1 (Cu,Zn-SOD) in the cytosol and mitochondrial intermembrane space, and SOD2 (Mn-SOD) in the mitochondrial matrix. They catalyze the conversion of superoxide to hydrogen peroxide. Despite having the same function as SOD2, the SOD1 enzyme was not found to be altered in RTD2 motoneurons compared with Ctrl motoneurons. These data suggest that ROS species may be more abundant in mitochondria, which indeed appear damaged, as described in Colasuonno et al. (2020b). SOD2 is also associated with the GPX3 activity, a seleno-protein with antioxidant potential that catalyzes the reduction of hydrogen peroxide, hydroperoxides, and lipid hydroperoxides by reduced glutathione (GSH). GSH plays an important role at the neuronal level as it is a free radical scavenger, redox modulator of ionotropic receptor activity and a neurotransmitter (Bains and Shaw, 1997). In RTD2 motoneurons, also *GPX3* mRNA levels were increased when compared to those of Ctrl motoneurons (Figure 5I). Moreover, we discovered out an altered NRF2/HO-1 pathway in RTD2 motoneurons, thus providing a possible explanation for the imbalance of the redox status already observed in these cells (Colasuonno et al., 2020b; Niceforo et al., 2021). Indeed HO-1, whose levels were significantly decreased in all RTD2 motoneurons (Figure 5J), plays a role in attenuating the production of ROS, as well as in reducing secondary endoplasmic reticulum (ER) stress and neuronal apoptosis (Xu et al., 2020). It should be noted that ER is the main intracellular calcium storage (Koch, 1990), and interestingly, RTD2 motoneurons have impaired calcium influx (Niceforo et al., 2021). Hence, the decreased levels of *H0-1* mRNA may promote ER stress, thus leading to altered release of calcium, impairment of calcium signaling and neuronal functionality in RTD2 motoneurons.

Importantly, the RTD2 cell type that showed the highest significantly altered gene expression levels were the motoneurons, while the analyses of *SOD1, SOD2, GPX3, HO-1* and *xCT* expression levels in Ctrl and RTD2 iPSCs and astrocytes (Figure 5) did not show statistical differences.

Altogether, the data collected on the expression of genes involved in the regulation of antioxidant defenses suggest the presence of increased oxidative stress in RTD2 compared to Ctrl motoneurons. Importantly, while RTD2 motoneurons displayed altered patterns of mRNA levels of genes relevant to respond to oxidative stress, RTD2 astrocytes showed mostly uncompromised mRNA levels of the same genes.

## 5 Conclusion

**Figure 6:**
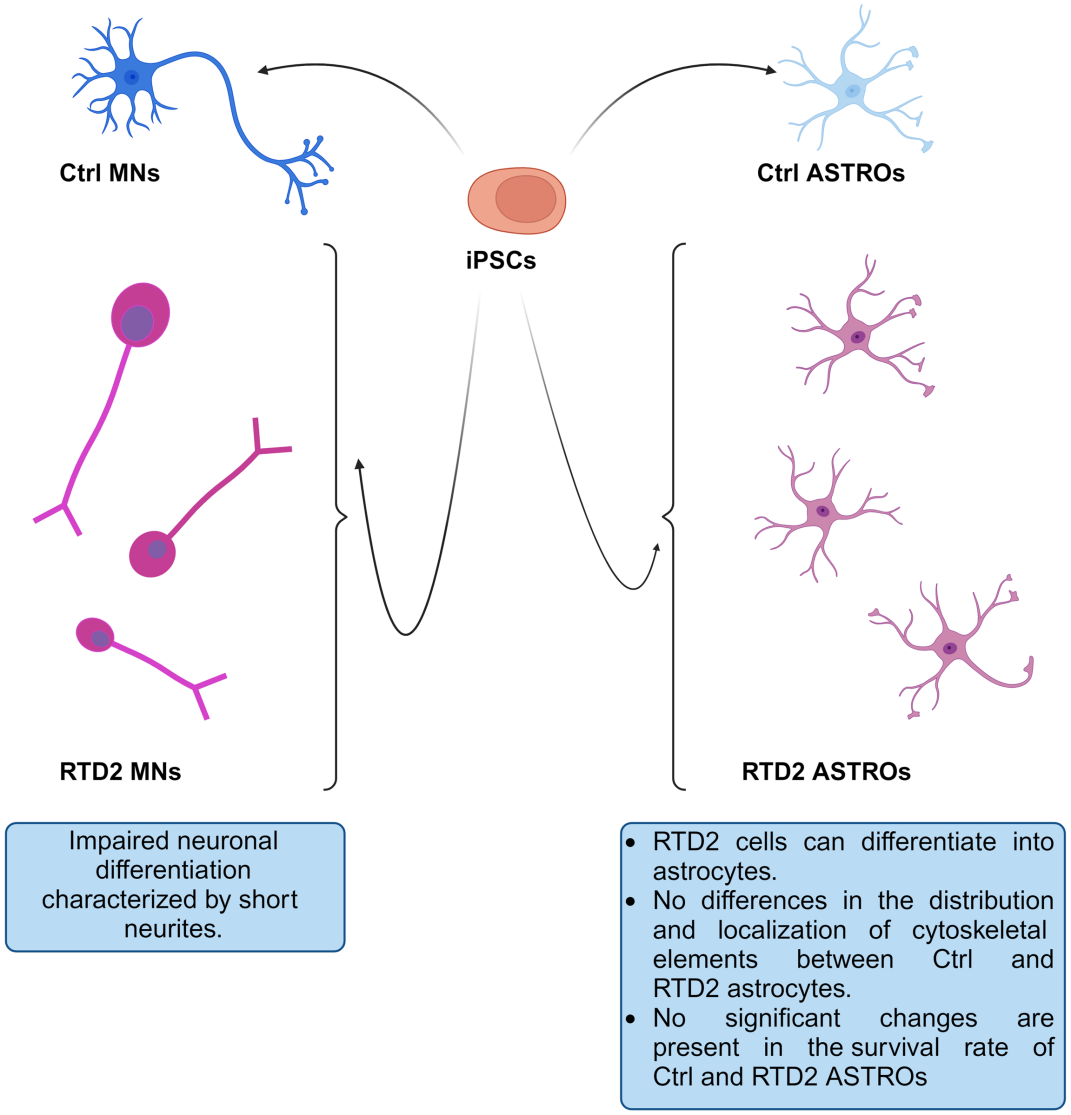
Schematic drawing depicting Ctrl and RTD2 MNs and ASTROs. In particular, RTD2 MNs show aberrant neuronal differentiation: shorter neurites, while RTD2 ASTROs do not show cytoskeletal alterations nor changes in their survival rate compared to Ctrl ASTROs.

In the present study, we used patient-derived iPSCs to better study and investigate RTD2 pathogenesis focusing on a previously unstudied cell type, the astrocytes. Exploiting this model, we deepened some new aspects of RTD2 pathology. In particular, we confirmed the ability of Ctrl and RTD2 iPSCs to differentiate into astrocytes. The data indicate that RTD2 astrocytes do not show altered features in terms of morphology, cell death nor viability compared to Ctrl astrocytes (Figure 6). Furthermore, by analyzing the mRNA levels of genes that regulate the redox state, we found an alteration in RTD2 iPSCs, and especially in motoneurons when compared to Ctrl cells. We also discovered that RTD2 astrocytes are similar to those derived from healthy individuals, suggesting that they are not affected by alterations of *SLC52A2* in terms of antioxidant gene expression. Noteworthy, the most relevant difference we observed in astrocytes concerns the expression of *RFVT2*. In RTD2 astrocytes, mRNA levels of this transporter are reduced by about half compared to control astrocytes. Additional research is required to clarify this aspect and gain a better understanding of the role played by astrocytes in RTD2, because the current data suggest that the motoneuron is the most affected cell type in RTD2 pathology.

## Data availability statement

The original contributions presented in the study are included in the article/Supplementary material, further inquiries can be directed to the corresponding author/s.

## Ethics statement

The studies involving humans were approved by Comitato Etico Ospedale Bambino Gesù. The studies were conducted in accordance with the local legislation and institutional requirements. Written informed consent for participation in this study was provided by the participants’ legal guardians/next of kin.

## Author contributions

VM: Data curation, Formal analysis, Funding acquisition, Investigation, Methodology, Software, Validation, Visualization, Writing – original draft. AL: Methodology, Writing – review & editing. EA: Methodology, Resources, Supervision, Writing – review & editing. LT: Data curation, Methodology, Writing – review & editing. VD’E: Data curation, Methodology, Writing – review & editing. VD’O: Data curation, Methodology, Writing – review & editing. SP: Data curation, Methodology, Writing – review & editing. MC: Data curation, Writing – review & editing. KM: Data curation, Resources, Writing – review & editing. MT: Data curation, Funding acquisition, Resources, Writing – review & editing. EB: Data curation, Funding acquisition, Resources, Writing – review & editing. TP: Data curation, Funding acquisition, Methodology, Project administration, Resources, Supervision, Writing – review & editing. CC: Conceptualization, Data curation, Funding acquisition, Project administration, Supervision, Writing – review & editing, Resources.

## Supplementary material

Supplementary Figure S1 Sanger sequencing of Ctrl and two (newly reprogrammed) RTD2 iPSCs. Electropherograms show the pathogenic *SLC52A2* mutations identified in Pt2 (a), and Pt3 (b), while the mutations carried by the iPSCs derived from Pt have been already reported in previous studies (Niceforo et al., 2021, Ref 33). The arrows indicate the position of the mutation.

## References

[ref1] Bains J. S. Shaw C. A. (1997). Neurodegenerative disorders in humans: the role of glutathione in oxidative stress-mediated neuronal death. Brain Res. Brain Res. Rev. 25, 335–358. doi: 10.1016/s0165-0173(97)00045-3, PMID: 9495562

[ref2] Barile M. Giancaspero T. A. Brizio C. Panebianco C. Indiveri C. Galluccio M.. (2013). Biosynthesis of flavin cofactors in man: implications in health and disease. Curr. Pharm. Des. 19, 2649–2675. doi: 10.2174/1381612811319140014, PMID: 23116402

[ref3] Barile M. Giancaspero T. A. Leone P. Galluccio M. Indiveri C. (2016). Riboflavin transport and metabolism in humans. J. Inherit. Metab. Dis. 39, 545–557. doi: 10.1007/s10545-016-9950-0 27271694

[ref4] Barres B. A. (2008). The mystery and magic of glia: a perspective on their roles in health and disease. Neuron 60, 430–440. doi: 10.1016/j.neuron.2008.10.013, PMID: 18995817

[ref5] Baxter P. S. Hardingham G. E. (2016). Adaptive regulation of the brain's antioxidant defences by neurons and astrocytes. Free Radic. Biol. Med. 100, 147–152. doi: 10.1016/j.freeradbiomed.2016.06.027 27365123 PMC5145800

[ref6] Binder D. K. Steinhäuser C. (2021). Astrocytes and epilepsy. Neurochem. Res. 46, 2687–2695. doi: 10.1007/s11064-021-03236-x 33661442

[ref7] Brown Charles Henry M. D. (1894). Infantile amyotrophic lateral sclerosis of the family type. J. Nerv. Ment. Dis. 19, 707–716.

[ref8] Ciccolella M. Catteruccia M. Benedetti S. Moroni I. Uziel G. Pantaleoni C.. (2012). Brown-Vialetto-van Laere and Fazio-Londe overlap syndromes: a clinical, biochemical and genetic study. Neuromuscul. Disord. 22, 1075–1082. doi: 10.1016/j.nmd.2012.05.007, PMID: 22824638

[ref9] Colasuonno F. Bertini E. Tartaglia M. Compagnucci C. Moreno S. (2020b). Mitochondrial abnormalities in induced pluripotent stem cells-derived motor neurons from patients with riboflavin transporter deficiency. Antioxidants (Basel, Switzerland) 9:1252. doi: 10.3390/antiox9121252, PMID: 33317017 PMC7763948

[ref10] Colasuonno F. Marioli C. Tartaglia M. Bertini E. Compagnucci C. Moreno S. (2022). New insights into the neurodegeneration mechanisms underlying riboflavin transporter deficiency (RTD): involvement of energy Dysmetabolism and cytoskeletal derangement. Biomedicines 10:1329. doi: 10.3390/biomedicines10061329, PMID: 35740351 PMC9219947

[ref11] Colasuonno F. Niceforo A. Marioli C. Fracassi A. Stregapede F. Massey K.. (2020a). Mitochondrial and Peroxisomal alterations contribute to energy Dysmetabolism in riboflavin transporter deficiency. Oxidative Med. Cell. Longev. 2020, 6821247–6821219. doi: 10.1155/2020/6821247, PMID: 32855765 PMC7443020

[ref12] Corti S. Nizzardo M. Simone C. Falcone M. Nardini M. Ronchi D.. (2012). Genetic correction of human induced pluripotent stem cells from patients with spinal muscular atrophy. Sci. Transl. Med. 4:165ra162. doi: 10.1126/scitranslmed.3004108, PMID: 23253609 PMC4722730

[ref13] Dermitzakis E. T. Clark A. G. (2002). Evolution of transcription factor binding sites in mammalian gene regulatory regions: conservation and turnover. Mol. Biol. Evol. 19, 1114–1121. doi: 10.1093/oxfordjournals.molbev.a004169 12082130

[ref14] D’Ezio V. Colasanti M. Persichini T. (2021). Amyloid-β 25-35 induces neurotoxicity through the up-regulation of astrocytic system xc. Antioxidants (Basel, Switzerland) 10:1685. doi: 10.3390/antiox10111685, PMID: 34829555 PMC8615014

[ref15] Espinós C. Galindo M. I. García-Gimeno M. A. Ibáñez-Cabellos J. S. Martínez-Rubio D. Millán J. M.. (2020). Oxidative stress, a crossroad between rare diseases and neurodegeneration. Antioxidants (Basel, Switzerland) 9:313. doi: 10.3390/antiox9040313, PMID: 32326494 PMC7222183

[ref16] Fakhoury M. (2018). Microglia and astrocytes in Alzheimer's disease: implications for therapy. Curr. Neuropharmacol. 16, 508–518. doi: 10.2174/1570159X15666170720095240, PMID: 28730967 PMC5997862

[ref17] Fox I. J. Paucar A. A. Nakano I. Mottahedeh J. Dougherty J. D. Kornblum H. I. (2004). Developmental expression of glial fibrillary acidic protein mRNA in mouse forebrain germinal zones--implications for stem cell biology. Brain Res. Dev. Brain Res. 153, 121–125. doi: 10.1016/j.devbrainres.2004.07.011 15464225

[ref18] Fransen M. Nordgren M. Wang B. Apanasets O. (2012). Role of peroxisomes in ROS/RNS-metabolism: implications for human disease. Biochim. Biophys. Acta 1822, 1363–1373. doi: 10.1016/j.bbadis.2011.12.001, PMID: 22178243

[ref19] Fujimura M. Yamamoto S. Murata T. Yasujima T. Inoue K. Ohta K. Y.. (2010). Functional characteristics of the human ortholog of riboflavin transporter 2 and riboflavin-responsive expression of its rat ortholog in the small intestine indicate its involvement in riboflavin absorption. J. Nutr. 140, 1722–1727. doi: 10.3945/jn.110.128330, PMID: 20724488

[ref20] Green P. Wiseman M. Crow Y. J. Houlden H. Riphagen S. Lin J. P.. (2010). Brown-Vialetto-Van Laere syndrome, a ponto-bulbar palsy with deafness, is caused by mutations in c20orf54. Am. J. Hum. Genet. 86, 485–489. http://doi:10.1016/j.ajhg.2010.02.006. doi: 10.1016/j.ajhg.2010.02.006, PMID: 20206331 PMC2833371

[ref21] Ilieva H. Polymenidou M. Cleveland D. W. (2009). Non-cell autonomous toxicity in neurodegenerative disorders: ALS and beyond. J. Cell Biol. 187, 761–772. doi: 10.1083/jcb.200908164, PMID: 19951898 PMC2806318

[ref22] Jaeger B. Bosch A. M. (2016). Clinical presentation and outcome of riboflavin transporter deficiency: mini review after five years of experience. J. Inherit. Metab. Dis. 39, 559–564. doi: 10.1007/s10545-016-9924-2, PMID: 26973221 PMC4920840

[ref23] John Lin C. C. Yu K. Hatcher A. Huang T. W. Lee H. K. Carlson J.. (2017). Identification of diverse astrocyte populations and their malignant analogs. Nat. Neurosci. 20, 396–405. doi: 10.1038/nn.4493, PMID: 28166219 PMC5824716

[ref24] Koch G. L. (1990). The endoplasmic reticulum and calcium storage. Bioessays 12, 527–531. doi: 10.1002/bies.950121105 2085319

[ref25] Lanciotti A. Brignone M. S. Macioce P. Visentin S. Ambrosini E. (2021). Human iPSC-derived astrocytes: a powerful tool to study primary astrocyte dysfunction in the pathogenesis of rare Leukodystrophies. Int. J. Mol. Sci. 23:274. doi: 10.3390/ijms23010274, PMID: 35008700 PMC8745131

[ref26] Ma Q. (2013). Role of nrf2 in oxidative stress and toxicity. Annu. Rev. Pharmacol. Toxicol. 53, 401–426. doi: 10.1146/annurev-pharmtox-011112-140320, PMID: 23294312 PMC4680839

[ref27] Marioli C. Magliocca V. Petrini S. Niceforo A. Borghi R. Petrillo S.. (2020). Antioxidant amelioration of riboflavin transporter deficiency in Motoneurons derived from patient-specific induced pluripotent stem cells. Int. J. Mol. Sci. 21:7402. doi: 10.3390/ijms21197402, PMID: 33036493 PMC7582490

[ref28] Mastrantonio R. D'Ezio V. Colasanti M. Persichini T. (2019). Nrf2-mediated system xc- activation in Astroglial cells is involved in HIV-1 tat-induced neurotoxicity. Mol. Neurobiol. 56, 3796–3806. doi: 10.1007/s12035-018-1343-y, PMID: 30209772

[ref29] Miao L. St Clair D. K. (2009). Regulation of superoxide dismutase genes: implications in disease. Free Radic. Biol. Med. 47, 344–356. doi: 10.1016/j.freeradbiomed.2009.05.018, PMID: 19477268 PMC2731574

[ref30] Niceforo A. Marioli C. Colasuonno F. Petrini S. Massey K. Tartaglia M.. (2021). Altered cytoskeletal arrangement in induced pluripotent stem cells (iPSCs) and motor neurons from patients with riboflavin transporter deficiency. Dis. Model. Mech. 14:dmm046391. doi: 10.1242/dmm.046391 33468503 PMC7927654

[ref31] Odom D. T. Dowell R. D. Jacobsen E. S. Gordon W. Danford T. W. MacIsaac K. D.. (2007). Tissue-specific transcriptional regulation has diverged significantly between human and mouse. Nat. Genet. 39, 730–732. doi: 10.1038/ng2047, PMID: 17529977 PMC3797512

[ref9001] Okita K. Matsumura Y. Sato Y. Okada A. Morizane A. Okamoto S.. (2011). A more efficient method to generate integration-free human iPS cells. Nature methods, 8, 409–412. doi: 10.1038/nmeth.1591, PMID: 21460823

[ref32] Rizzo F. Ramirez A. Compagnucci C. Salani S. Melzi V. Bordoni A.. (2017). Genome-wide RNA-seq of iPSC-derived motor neurons indicates selective cytoskeletal perturbation in Brown-Vialetto disease that is partially rescued by riboflavin. Sci. Rep. 7:46271. doi: 10.1038/srep46271, PMID: 28382968 PMC5382781

[ref33] Rossi S. Cozzolino M. (2021). Dysfunction of RNA/RNA-binding proteins in ALS astrocytes and microglia. Cells 10:3005. doi: 10.3390/cells10113005, PMID: 34831228 PMC8616248

[ref34] Santello M. Volterra A. (2010). Neuroscience: Astrocytes as aide-mémoires. Nature 463, 169–170. doi: 10.1038/463169a, PMID: 20075911

[ref35] Su Z. Z. Leszczyniecka M. Kang D. C. Sarkar D. Chao W. Volsky D. J.. (2003). Insights into glutamate transport regulation in human astrocytes: cloning of the promoter for excitatory amino acid transporter 2 (EAAT2). Proc. Natl. Acad. Sci. U. S. A. 100, 1955–1960. doi: 10.1073/pnas.0136555100, PMID: 12578975 PMC149940

[ref36] Suwannasom N. Kao I. Pruß A. Georgieva R. Bäumler H. (2020). Riboflavin: the health benefits of a forgotten natural vitamin. Int. J. Mol. Sci. 21:950. doi: 10.3390/ijms21030950, PMID: 32023913 PMC7037471

[ref37] Takahashi S. Mashima K. (2022). Neuroprotection and disease modification by astrocytes and microglia in Parkinson disease. Antioxidants (Basel, Switzerland) 11:170. doi: 10.3390/antiox11010170, PMID: 35052674 PMC8773262

[ref38] Tanaka J. Toku K. Zhang B. Ishihara K. Sakanaka M. Maeda N. (1999). Astrocytes prevent neuronal death induced by reactive oxygen and nitrogen species. Glia 28, 85–96. doi: 10.1002/(sici)1098-1136(199911)28:2<85::aid-glia1>3.0.co;2-y, PMID: 10533053

[ref39] Trigo D. Avelar C. Fernandes M. Sá J. da Cruz E. Silva O. (2022). Mitochondria, energy, and metabolism in neuronal health and disease. FEBS Lett. 596, 1095–1110. doi: 10.1002/1873-3468.14298 35088449

[ref40] Van Laere J. (1966). Paralysie bulbo-pontine chronique progressive familiale avec surdité. Un cas de syndrome de Klippel-Trenaunay dans la même fratrie. Problèmes diagnostiques et génétiques [Familial progressive chronic bulbo-pontine paralysis with deafness. A case of Klippel-Trenaunay syndrome in siblings of the same family. Diagnostic and genetic problems]. Rev. Neurol. 115, 289–295, PMID: 5969547

[ref41] Verkhratsky A. Nedergaard M. (2018). Physiology of Astroglia. Physiol. Rev. 98, 239–389. doi: 10.1152/physrev.00042.2016, PMID: 29351512 PMC6050349

[ref42] Vialetto E. (1936). Contributo alla forma ereditaria della paralisi bulbare progressive. Riv. Sper. Fren. 40, 1–24.

[ref43] Xu B. Qin Y. Li D. Cai N. Wu J. Jiang L.. (2020). Inhibition of PDE4 protects neurons against oxygen-glucose deprivation-induced endoplasmic reticulum stress through activation of the Nrf-2/HO-1 pathway. Redox Biol. 28:101342. doi: 10.1016/j.redox.2019.101342, PMID: 31639651 PMC6807264

[ref44] Yan Y. Shin S. Jha B. S. Liu Q. Sheng J. Li F.. (2013). Efficient and rapid derivation of primitive neural stem cells and generation of brain subtype neurons from human pluripotent stem cells. Stem Cells Transl. Med. 2, 862–870. doi: 10.5966/sctm.2013-0080, PMID: 24113065 PMC3808201

